# Secure attachment priming protects against relapse of fear in Young adults

**DOI:** 10.1038/s41398-021-01715-x

**Published:** 2021-11-13

**Authors:** Metaxia Toumbelekis, Belinda J. Liddell, Richard A. Bryant

**Affiliations:** University of New South Wales, Sydney, Australia

**Keywords:** Physiology, Psychiatric disorders, Human behaviour

## Abstract

Previous studies have shown that activating the attachment system attenuates fear learning. This study aimed to explore whether attachment priming can also impact on fear extinction processes, which underpin the management of anxiety disorders. In this study, 81 participants underwent a standard fear conditioning and extinction protocol on day 1 and returned 24 h later for an extinction recall and reinstatement test. Half the participants were primed to imagine their closest attachment figure prior to undergoing extinction training, while the other half were instructed to imagine a positive situation. Fear-potentiated startle and subjective expectancies of shock were measured as the primary indicators of fear. Attachment priming led to less relapse during the reinstatement test at the physiological but not subjective levels. These findings have translational potential to imply that activating awareness of attachment figures might augment long-term safety memories acquired in existing treatments to reduce relapse of fear.

Anxiety disorders are among the most common mental health disorders worldwide, and incur enormous burdens to individuals, families, communities, and health systems [1]. The best available treatment of anxiety typically involve exposure to reminders of perceived threat; however, there is a substantial proportion of people who do not respond to this approach [2]. For example, one meta-analysis estimates remission rates of anxiety following CBT treatment at only 48% [3]. Moreover, even where individuals improve the following treatment, relapse of fear is common [4]. Accordingly, there is a need to explore ways to augment exposure therapy to achieve better treatment response.

To enable the enhancement of exposure processes, there is a need to investigate potential strategies that can augment the mechanisms underlying how fear can be inhibited. The traditional model to understand these processes has been that of fear learning [5], which involves training participants in an experimental setting to fear benign stimuli by the association of the stimuli with an aversive outcome (e.g. a mild electric shock). The learned fear can be subsequently extinguished by repeated presentations of the ‘conditioned stimulus’ (CS) in the absence of the aversive outcome (i.e. extinction learning). This paradigm has been essential in modeling relapse of anxiety following the passage of time (spontaneous recovery), a change in environmental context (renewal) or a new stressful event (reinstatement) [6].

There have been numerous pharmacological and direct stimulation approaches to augment exposure by targeting extinction mechanisms; however, these have yielded only modest success [7]. One potential method to augment extinction may be by enhancing people’s awareness of their attachment figures. Priming attachment awareness involves activating mental accessibility of one’s attachment figures, either by their physical proximity, visual images or even mentally visualizing them [8]. Attachment primes have been found to reduce the acquisition and consolidation of fear [9–11], and enhance extinction learning [12]. Regarding the latter study, participants were presented with visual images of participants’ attachment figures (relative to images of strangers) during each trial in extinction learning; this procedure reduced relapse of fear immediately the following extinction and at a fear reinstatement test a day later.

The current study aimed to address two features that must be addressed before it can be concluded that attachment priming has specific value for augmenting extinction learning. First in contrast to the Hornstein et al. (2017) [12] study, a more ecologically valid attachment prime is needed that can be translated to practical settings because it is not feasible to have individual’s attachment figures physically or imaginally present during each and every extinction learning experience. Therefore, we utilized a visual imagery task as a method to activate brief awareness of one’s attachment figure immediately prior to extinction learning. This procedure could be readily applied to real-world settings in which people approach feared stimuli to overcome anxiety. For example, if a therapist could be considered as an important attachment figure, then building a strong therapeutic relationship prior to undergoing exposure therapy could improve therapeutic outcomes. Second, previous studies have shown that presenting positive stimuli [13, 14] or positive mood induction paradigms [15] prior to undergoing fear extinction reduces negative evaluations of the conditioned stimulus, inhibits the fear response and can enhance extinction learning and reduce relapse. Therefore, it is of primary importance to isolate whether the benefit of priming attachment during extinction learning is due to increased positive mood or the specific priming of attachments.

To address these issues, we assessed extinction learning and retention after activating brief awareness of an attachment figure prior to the extinction learning, and compared this to a positive mood induction to determine the unique effects of attachment priming on enhancing extinction learning and reduce return of fear. We hypothesized that the attachment priming group would demonstrate faster within-session extinction, between session extinction and show less reinstatement of fear. However, we anticipated that these effects may be moderated by important individual differences. There is much evidence that attachment priming is most effective for those who have a more secure attachment style [11, 16, 17] because people who are either anxiously or avoidantly attached do not have a secure base from which to draw from, and accordingly they do not receive the same benefits of attachment proximity [8].

## Methods

### Participants

**Fig. 1:**
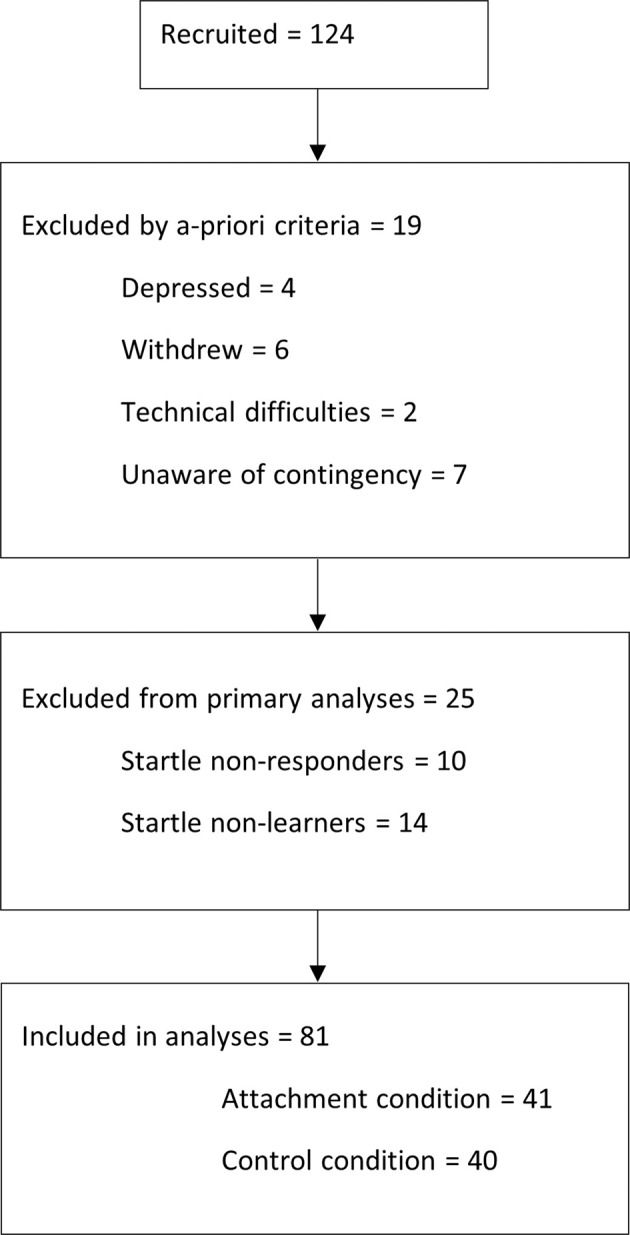
Consort diagram of participants excluded. Participants excluded because of withdrawal, depression, technical difficulties, non-awareness of contingencies, and non-startler esponse.

Participants were 124 undergraduate psychology students (86 females; mean age = 19.82 years, SD = 3.13) who participated in return for payment (at a rate of $20 per hour, totaling to $AUD60) or received course credit for their participation. All procedures were approved by the Human Research Ethics Committee at the University of New South Wales (HC15507), and participants provided written informed consent. Participants were randomly assigned to the attachment or positive control prime conditions by a computer-generated randomization software. Four participants were excluded from participating because they scored in the ‘Extremely Severe’ range on any of the Depression, Anxiety and Stress Scale (DASS [18]) subscales. Further additional participants were excluded from the final sample because a) they withdrew their consent during the study (one participant) or did not attend both sessions (five participants); b) there were technical difficulties that resulted in excessive missing data (two participants); d) they could not verbalize stimulus contingencies (seven participants); e) they met a priori criteria of basic fear learning and were classified as ‘startle non-responders’ (10 participants) or ‘startle non-learners’ (14 participants) as described below. The final sample consisted of 81 participants (see consort diagram in Fig. 1).

### Materials

#### Self-report questionnaires

The DASS-21 [18] was used to assess negative emotional states. This measure includes 21 items that comprise subscales of depression, anxiety and stress. These subscales have good internal consistencies of 0.94, 0.87 and 0.91, respectively [19]. The Experiences in Close Relationships questionnaire (ECR [20]) was used to assess individual differences in attachment style. This questionnaire consists of 36 items that measure attachment anxiety and attachment avoidance. It has good internal consistency of 0.94 and 0.93 for the two subscales of anxiety and avoidance, respectively [21]. The Vividness of Visual Imagery Questionnaire (VVIQ [22]) measures an individual’s capacity to elicit mental visual images, and was administered to index the comparability of participants’ capacities in both prime conditions to imagine the prescribed primes. One item of the VVIQ was altered because it asked participants to visualize a “relative or friend whom you frequently see”; as this may have elicited an attachment figure, this item was modified to “think of a person you see often but don’t know personally (e.g. a barista at your local cafe)”. Finally, participants also completed a demographics and health questionnaire that asked them to report on their gender, age, years of education, broad ethnic background, country of birth, languages spoken at home, any medical conditions or medications they take regularly, their weight and height.

#### Stimulus delivery

Participants sat approximately 1 meter in front of a video display monitor, and were told to pay attention to the screen at all times. Stimuli were presented onto a black screen with a white fixation cross in the center. The visual stimuli used as conditioned stimuli (CS’s) were colored squares (orange or purple) presented in the center of the screen for eight seconds. Colors of squares were counterbalanced across participants such that half received the orange square as the CS+ and the purple square as the CS−, and the other half vice versa. Auditory stimuli used as the startle probes were 40 ms bursts of white noise measuring at 100 dB, with near instantaneous rise-time and presented through binaural headphones. These were presented during each CS (either six or seven seconds after the onset of the CS) and within half of the inter-trial intervals (ITIs). The startle probes occurring within the ITI (named startle-alone trials hereafter) were presented at a varied time, with the constraint that they would not occur within the eight seconds preceding or following a CS trial [23]. There was a constant background pink-noise set at 65 dB. The stimulus delivery software used was Presentation® (Neurobehavioral Systems, Inc.).

The unconditioned stimulus was a mild electric stimulation (500 ms in duration) delivered to the left forearm of each participant. This was delivered through a stimulating bar electrode with 9 mm disks separated by 30 mm (ADInstruments). The voltage was constant and set at 5 V, with the level of the amperes titrated to each participant from a minimum of 0.3 mA to a maximum of 30 mA, up to the point where it became “uncomfortable but not yet painful”. The actual range of levels chosen by participants varied from 0.3 mA to 9.6 mA (SD = 1.90).

#### Prime instructions

The mental visualization protocol for the attachment prime asked participants to think of a “specific person in your life who is currently very supportive to you, who you could depend on and turn to for help, and who makes you feel safe and loved.” Participants then answered some questions on their relation to this person, and described a typical interaction with them.

The control group was required to identify a “specific hypothetical situation that would make you very happy, something that would fulfill all your dreams or make your life easier or more pleasant, but something that has nothing to do with anybody else, so this situation must only involve yourself.” They were prompted to modify their choice if it was considered by the experimenter (using their clinical judgment) to have a social or attachment-related aspect. The situations chosen tended to be based on a sense of achievement, wealth or fame. Control participants then answered questions providing details of the situation they chose and how they would react to the situation.

All participants provided ratings on how happy the chosen person/situation made them feel, how excited, and their level of closeness with the person/ others in the situation on 10-point Likert scales (0 = *“not at all happy/excited/close”*, 9 = “*very happy/excited/close”*). They were then instructed to vividly imagine the prime for three minutes with their eyes closed, with prompts to *“continue thinking of the person/situation”* given every minute, and finally rated the vividness of the mental image (0 = “*not at all vivid”*, 5 = *“very vivid*”).

### Measures

#### Startle

The acoustic eyeblink startle reflex was measured by recording electromyography (EMG) activity of the orbicularis oculi muscle in response to the startle probe described above. Methods followed the suggestions from the Committee Report by Blumenthal et al. [24]. See Supplementary Methods for more details.

#### Expectancy of shock ratings

During each CS trial, the participants were required to provide a rating of their expectancy of shock on a 1–10 scale (1 = “certain no shock”, 5 = “uncertain”, 10 = “certain shock”) using a sliding bar on a response meter (MLT1601/ST, ADInstruments, Sydney).

### Procedure

A differential fear conditioning and fear extinction protocol was adapted from the protocol used by Grillon and Ameli [25]. The study included the following phases: startle habituation, pre-conditioning, conditioning, extinction, extinction recall, reinstatement (see below and see Supplementary Methods for full details).

Following guideline recommendations [23, 24], nine startle probes were delivered before any other part of the fear learning protocol. Then shock electrodes were attached and the level titrated as described above.

Participants were given instructions prior to beginning pre-conditioning implying that shocks may or may not be delivered on each trial. The pre-conditioning phase included four non-reinforced trials of the CS+ and CS− [23].

The conditioning phase included 16 trials of each of the CS+ and CS−, where a pseudo-random 10 or those 16 CS+ trials were paired with the US (shock), while the CS− trials were never paired with shock. The order of each trial was randomly generated with the constraint that no more than four of the same trial could be presented consecutively. Also, no more than four reinforced or non-reinforced CS+ trials could occur in a row, to minimize the possibility of any rapid extinction learning to occur prematurely within conditioning.

There was a 25-minute break prior to the extinction phase, during which participants watched a neutral film clip about airplanes and completed questionnaires. In the final 5 minutes, the attachment or control prime was administered, followed immediately by extinction, which was identical to conditioning with the exception that no US’s were delivered.

Participants returned 24 h later for part 2 of the study. The first phase was an extinction recall test, identical to extinction but with only 12 trials of each of the CS+ to CS−, to avoid excessive extinction learning. A fear reinstatement test followed a five-minute break. Immediately prior to this phase, four unsignalled US’s were delivered [26]. Following this, a further 12 CS+ and 12 CS− trials (non-reinforced) were presented. At the conclusion of this, participants were debriefed.

### Data analysis

We calculated the required sample size on the basis of a prior study of fear acquisition following attachment priming [10]. Using the parameters of this prior study, the power analysis indicated that to identify an effect of attachment priming on subsequent fear recall, we would require at least 42 participants in each condition (90% power, α = 0.05, two-tailed). We oversampled to achieve a total sample of at least 84 participants allowing for exclusions due to technical issues, drop-outs, and failing to learn contingencies.

For each block of trials, scores were averaged across four trials, as is common practice in human fear conditioning paradigms [27]. Participants were classified as startle ‘non-responders’ if they had three or more consecutive missing or 0 responses during a baseline phase, including habituation, pre-conditioning or startle-alone trials. Fear-potentiated startle (FPS) was defined as the differential startle response to the CS+ compared to the CS−. Participants who failed to show a fear-potentiated startle effect during acquisition (where CS+ was greater than CS−) were also excluded as startle ‘non-learners’. This was an a priori decision in order to only assess fear extinction and fear memory for participants for whom fear learning could reliably be established. Further post-hoc analyses including these participants classified as ‘non-learners’ are included in the Supplementary Methods.

Startle and expectancy data in each phase of the fear learning protocol were analyzed via separate repeated measures ANOVAs. To test the hypothesis that the attachment prime will affect fear learning and/or memory, a Group (attachment vs. positive control) by CS (CS+ vs. CS−) by block ANOVA was performed on each phase.

To test for potential moderating effects of attachment style, first fear recall scores were calculated for each participant as percentages of their overall levels of fear-potentiated startle (FPS; CS+minus CS−) during the extinction, recall and reinstatement phases proportional to their overall FPS during acquisition. Then linear multiple regressions were conducted adding the group variable, scores on the ECR attachment anxiety or attachment avoidance subscales, and the interaction of those scores with their group. The dependent variable was the percent recall score.

## Results

### Participant characteristics

After exclusion criteria were applied (see Fig. 1), the final sample consisted of 81 participants [57 females, mean age = 19.79 (SD = 1.81)], with 41 participants in the attachment group and 40 in the control group. Groups were equally distributed on demographic characteristics. There were no pre-existing differences between groups on anxiety, depression, capacity for visual imagery, or trait levels of attachment security (See Table 1). For a summary of these analyses including the participants excluded as ‘non-learners’, see the Supplementary Methods and Results (Table S1, Table S2). In summary, no differences in the patterns of results were found, with the exception of the analyses on percent recall scores. Post-hoc analyses further showed that the participants excluded as ‘non-learners’ were not differentiating between CS+ and CS− in their startle scores across any of the phases in the experiment, nor where they responding proportionally different to the CS− than the CS+ or startle-alone baseline trials as might be expected if they were showing generalized fear.

**Table 1:** Participant characteristics broken down into means and standard deviations for each group.

Measure	Attachment		Positive Control			
	*M*	SD	*M*	SD	*F* (1,79)	*p*
Age	19.87	1.91	19.70	1.73	0.21	0.649
Years of Education	14.35	1.46	13.79	1.48	2.51	0.117
DASS (Depression)	2.83	3.29	2.68	3.17	0.06	0.804
DASS (Anxiety)	2.68	2.94	2.25	2.32	0.48	0.490
DASS (Stress)	4.43	3.49	3.85	2.56	0.89	0.350
VVIQ	3.71	0.65	3.66	0.67	0.01	0.922
ECR (Anxiety)	3.31	0.98	3.40	1.04	0.03	0.856
ECR (Avoidance)	2.83	1.07	2.88	1.08	<0.001	0.992

*DASS* depression, anxiety, stress scale. *VVIQ* vividness of visual imagery questionnaire, *ECR* experiences in close relationship scale.

One-way ANOVA statistics (*F(1,79)* and *p-*values) also demonstrate statistically there are no differences in demographic variables between the groups.

### Subjective ratings of the prime

The attachment and control prime were rated equally in respect to positive mood (*F*(1,79) = 1.16, *p* = 0.284, *η*^*2*^_*p*_ = 0.014), while the control prime was rated as more “exciting” (*F*(1,79) = 4.76, *p* = 0.032, *η*^*2*^_*p*_ = 0.057). The attachment prime was rated as eliciting closer feelings to others (*F*(1,79) = 64.55, *p* < 0.001, *η*^*2*^_*p*_ = 0.450) and as being more vivid (*F*(1,79) = 7.04, *p* = 0.010, *η*^*2*^_*p*_ = 0.082). See Table S3 and Fig. S1 in Supplementary Materials.

### Fear learning

**Fig. 2:**
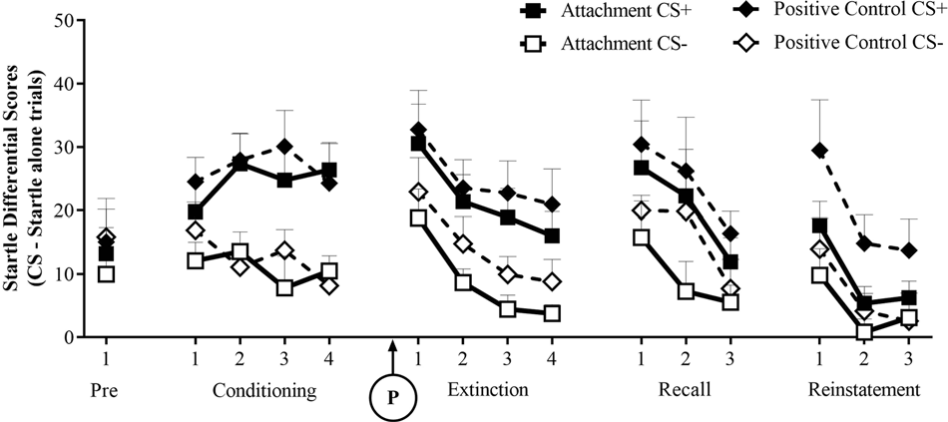
Startle (mean scores + SEMs to the CS+ and CS−) during pre-conditioning, conditioning, extinction, extinction recall and reinstatement phases, respectively. Note startle scores are graphed as differential scores (compared to startle-alone trials) and averaged across groups of 4 trials. The experimental manipulation, receiving the attachment or positive control imagery task, was delivered immediately prior to the extinction phase (‘P’ on the graph). Groups differed in their level of startle during the reinstatement phase.

**Fig. 3:**
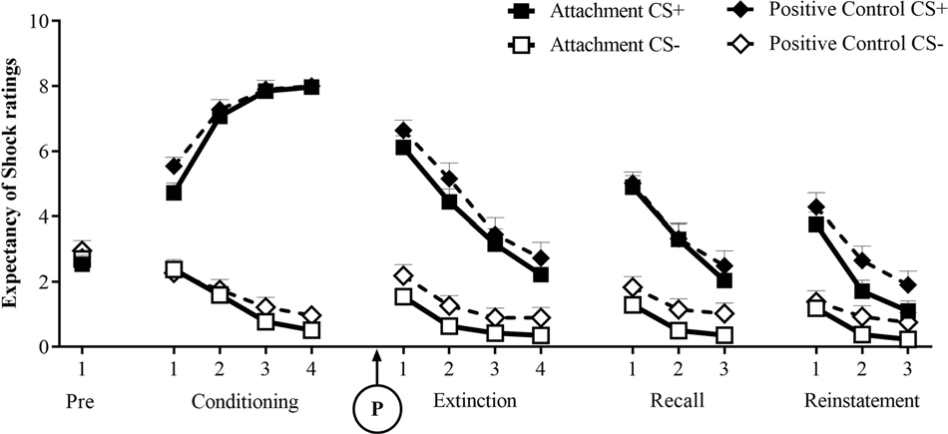
Subjective ratings of expectancy of shock (mean scores + SEMs to the CS+ and CS−) during pre-conditioning, conditioning, extinction, extinction recall and reinstatement phases, respectively. Note expectancy scores are averaged across groups of 4 trials. The experimental manipulation, receiving the attachment or positive control imagery task, was delivered immediately prior to the extinction phase (‘P’ on the graph). Groups did not significantly differ in their level of expectancy of shock.

Figures 2 and 3 outline the startle and expectancy scores, respectively, for each primed group across blocks of trials. Averaged across both groups, startle scores decreased across trials during habituation (*F*(1,79) = 67.47, *p* < 0.001, *η*^*2*^_*p*_ = 0.461). There were no differences in startle or expectancy ratings of shock to the CS+ and CS− during pre-conditioning [startle: *F*(1,79) = 0.73, *p* = 0.397, *η*^*2*^_*p*_ = 0.009; expectancy: *F*(1,79) = 0.86, *p* = 0.347, *η*^*2*^_*p*_ = 0.011].

During conditioning, there was a significant main effect of CS [startle: *F*(1,79) = 55.43, *p* < 0.001, *η*^*2*^_*p*_ = 0.412; expectancies: *F*(1,79) = 856.64, *p* < 0.001, *η*^*2*^_*p*_ = 0.916], where scores were greater for the CS + than the CS−, indicating successful acquisition of fear learning and fear-potentiated startle (FPS). Note however, that this result is inflated due to the exclusion of startle non-learners. There was also an increase in learning across blocks [CS x blocks interaction: startle: *F*(1,79) = 6.11, *p* = 0.016, *η*^*2*^_*p*_ = 0.072; expectancies: *F*(1,79) = 271.47, *p* < 0.001, *η*^*2*^_*p*_ = 0.775].

A significant main effect of CS was maintained across the extinction phase [startle: *F*(1,79) = 37.69, *p* < 0.001, *η*^*2*^_*p*_ = 0.323; expectancies *F*(1,79) = 146.59, *p* < 0.001, *η*^*2*^_*p*_ = 0.650], which extinguished across blocks only for expectancy scores [linear CS x blocks interaction: *F*(1,79) = 59.44, *p* < 0.001, *η*^*2*^_*p*_ = 0.429]. There was a reduction in fear to both the CS + and CS− across blocks in extinction for startle [*F*(1,79) = 26.50, *p* < 0.001, *η*^*2*^_*p*_ = 0.251].

The CS main effect remained in the extinction recall phase on day 2 [startle: *F*(1,79) = 21.72, *p* < 0.001, *η*^*2*^_*p*_ = 0.216; expectancies: *F*(1,79) = 95.28, *p* < 0.001, *η*^*2*^_*p*_ = 0.547]. Again this decreased linearly across blocks though only reaching a significant level for expectancy scores [CS × blocks interaction−expectancies: *F*(1,79) = 38.74, *p* < 0.001, *η*^*2*^_*p*_ = 0.329]. Again, startle scores decreased to both the CS+ and CS− [*F*(1,79) = 24.47, *p* < 0.001, *η*^*2*^_*p*_ = 0.236]. This indicates that there was extinction of both learned fear to the paired CS + cue as well as generalized fear to the safe, unpaired CS− cue.

However, even in the final block of trials within the extinction recall phase, there remained significantly greater levels of fear to the CS+ than the CS−, indicating that extinction learning was not complete [startle: *F*(1,79) = 9.15, *p* = 0.003, *η*^*2*^_*p*_ = 0.104; expectancies: *F*(1,79) = 32.78, *p* < 0.001, *η*^*2*^_*p*_ = 0.293].

From the last block during recall to the first block in reinstatement, there was an increase in fear to both the CS+ and CS− [main effect of phase: startle: *F*(1,79) = 11.32, *p* = 0.001, *η*^*2*^_*p*_ = 0.125; expectancies *F*(1,79) = 57.77, *p* < 0.001, *η*^*2*^_*p*_ = 0.422]. For expectancies, but not startle, this effect was greater for the CS+ than CS− [phase × CS interaction−startle: *F*(1,79) = 2.45, *p* = 0.122, *η*^*2*^_*p*_ = 0.030; expectancies: *F*(1,79) = 22.49, *p* < 0.001, *η*^*2*^_*p*_ = 0.222], indicating successful reinstatement of fear.

Also, across only the reinstatement phase, there was an overall main effect of CS [startle: *F*(1,79) = 27.71, *p* < 0.001*, η*^*2*^_*p*_ = 0.260; expectancies: *F*(1,79) = 58.83, *p* < 0.001*, η*^*2*^_*p*_ = 0.427]. This effect decreased linearly across blocks in expectancies but not FPS [CS x block interaction – startle: *F*(1,79) = 3.61, *p* = 0.061*, η*^*2*^_*p*_ = 0.044; expectancies: *F*(1,79) = 51.76, *p* < 0.001*, η*^*2*^_*p*_ = 0.396]. For startle, responses decreased to both the CS+ and CS− (main effect of block−startle: *F*(1,79) = 30.59, *p* < 0.001 *η*^*2*^_*p*_ = 0.279).

### Priming effects

As was expected, prior to the attachment manipulation, there were no differences between groups during startle habituation (*F*(1,79) = 1.08, *p* = 0.301, *η*^*2*^_*p*_ = 0.014) or in pre-conditioning [startle: *F*(1,79) = 0.35, *p* = 0.558, *η*^*2*^_*p*_ = 0.004; expectancy: *F*(1,79) = 0.52, *p* = 0.475, *η*^*2*^_*p*_ = 0.006]. There were also no significant differences between groups in the average FPS or expectancies during conditioning [CS × group interaction−startle: *F*(1,79) = 0.13, *p* = 0.716, *η*^*2*^_*p*_ = 0.002; expectancies: *F*(1,79) = 0.01, *p* = 0.920, *η*^*2*^_*p*_ < 0.001] or in the rate of learning [CS × Block × Group interaction−startle: *F*(1,79)<0.001, *p* = 0.997, *η*^*2*^_*p*_ < 0.001; expectancies: *F*(1,79) = 1.46, *p* = 0.231, *η*^*2*^_*p*_ = 0.018].

Examining within-session extinction effects, following the prime manipulation, the attachment and control groups demonstrated comparable levels of FPS and expectancies of shock across extinction [CS × group interaction−startle: *F*(1,79) = 0.08, *p* = 0.779, *η*^*2*^_*p*_ = 0.001; expectancies: *F*(1,79) = 0.014, *p* = 0.905, *η*^*2*^_*p*_ < 0.001]. Also, no differences were found in the rate of extinction for startle or expectancies [CS × block × group interaction−startle: *F*(1,79) = 0.42, *p* = 0.518, *η*^*2*^_*p*_ = 0.005; expectancies: *F*(1,79)<0.001, *p* = 0.987, *η*^*2*^_*p*_ < 0.001]. Similarly, examining between session extinction, no group differences were found in extinction recall on day 2 [CS × group interaction−startle: *F*(1,79) = 0.23, *p* = 0.633, *η*^*2*^_*p*_ = 0.003; expectancies: *F*(1,79) = 0.67, *p* = 0.416, *η*^*2*^_*p*_ = 0.008].

Looking at reinstatement effects, from the recall phase to the reinstatement phase, there was a significant phase by CS by group interaction [startle: *F*(1,79) = 4.30, *p* = 0.041*, η*^*2*^_*p*_ = 0.052; expectancies: *F*(1,79) = 4.46, *p* = 0.038*, η*^*2*^_*p*_ = 0.053].

Follow-up simple effects analyses for startle scores found that the control group demonstrated a greater increase of differential and overall fear from the end of recall to beginning of reinstatement [phase × CS interaction: *F*(1,39) = 1.49, *p* = 0.229*, η*^*2*^_*p*_ = 0.037; main effect of phase: *F*(1,39) = 7.75, *p* = 0.008*, η*^*2*^_*p*_ = 0.166] than the attachment group [phase x CS interaction: *F*(1,40) = 0.51, *p* = 0.479*, η*^*2*^_*p*_ = 0.013; main effect of phase: *F*(1,39) = 4.58, *p* = 0.039*, η*^*2*^_*p*_ = 0.103]. These simple effects did not hold for expectancy scores.

Also, the attachment group showed lower levels of fear overall—to both the CS+ and CS− during the reinstatement phase, which only reached significance for startle scores [group main effect – startle: *F*(1,79) = 4.24, *p* = 0.043*, η*^*2*^_*p*_ = 0.051; expectancies: *F*(1,79) = 2.68, *p* = 0.105*, η*^*2*^_*p*_ = 0.033]. This demonstrates that the attachment prime may have enhanced fear inhibition AND strengthened generalized safety learning as well.

**Fig. 4:**
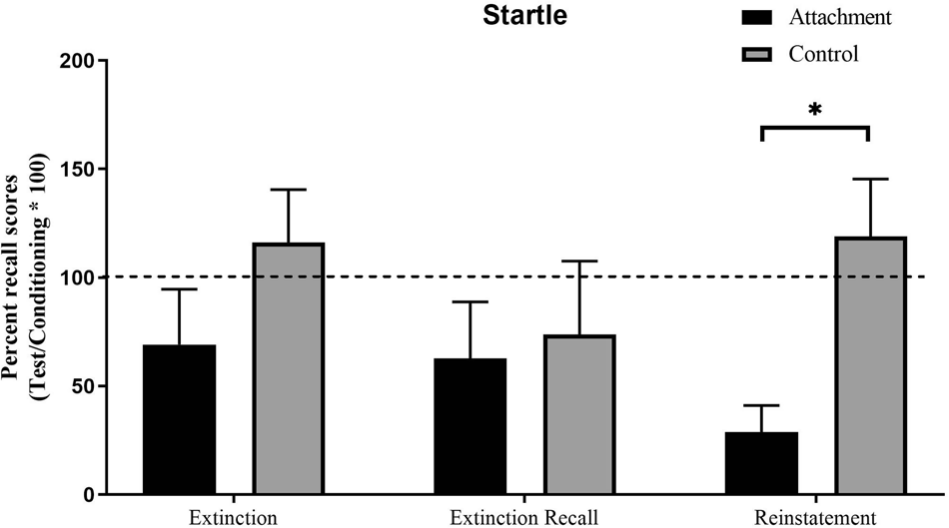
Percent recall scores of startle data across the three phases of extinction learning, extinction recall and reinstatement test. Recall scores were calculated for each participant by taking their average differential score (CS+ minus CS−) during the extinction, recall and reinstatement phases, dividing by their average differential score during fear conditioning and multiplying by 100.

When examining percent recall scores at the extinction, extinction recall and reinstatement phases (each relative to levels of conditioning), the only significant group differences emerged for startle scores at the level of the reinstatement test. Here, the attachment group demonstrated less reinstatement of fear than the control group (*F*(1,79) = 8.90, *p* = 0.004*, η*^*2*^_*p*_ = 0.102). See Fig. 4.

### Moderation analyses

There were no significant moderation effects of individual differences in attachment style on percent extinction, recall or reinstatement scores (all *p*’s > 0.05; see Table 2). Nor did any of the differences between groups on subjective ratings of the primes (valence, arousal, closeness or vividness) moderate the effect of prime on percent reinstatement scores (all *p*’s > 0.05). See Table S4 in Supplementary Materials.

**Table 2:** Moderation analyses on attachment style.

Dependent Variable	Source	*B*	*SE B*	*b*	*t*	*p*
Extinction	ECR Anxiety × Prime	−47.99	30.13	−0.88	−1.593	0.115
Extinction	ECR Avoidance × Prime	−31.93	27.937	−0.55	−1.143	0.257
Extinction recall	ECR Anxiety × Prime	22.93	43.764	0.29	0.524	0.602
Extinction recall	ECR Avoidance × Prime	−32.43	40.413	−0.39	−0.802	0.425
Reinstatement	ECR Anxiety × Prime	−15.75	30.064	−0.28	−0.524	0.602
Reinstatement	ECR Avoidance × Prime	−17.64	27.759	−0.29	−0.636	0.527

Extinction: ECR Anxiety, *R*^2^ = 0.099; ECR Avoidance, *R*^2^ = 0.048. Extinction recall: ECR Anxiety, *R*^2^ = 0.009; ECR Avoidance, *R*^2^ = 0.017. Reinstatement: ECR Anxiety, *R*^2^ = 0.119; ECR Avoidance, *R*^2^ = 0.119.

Hierarchical Linear Regression models conducted on percent extinction, recall and reinstatement scores separately as dependant variables and examining moderation effects of ECR anxiety or avoidance scores as continuous moderating variables. Tabled below are the regression coefficients for the interaction variable of the ECR scores with the group variable—showing no significant moderation effects of ECR scores on percent recall scores.

## Discussion

The current study investigated secure attachment priming effects on extinction learning and its long-term effects on relapse of fear. We found that the attachment group demonstrated less return of fear in a fear reinstatement test. This was demonstrated as both a reduction in the level of fear-potentiated startle to the paired CS+ as well as overall less fear shown to the safety cue, CS−. Unexpectedly, the attachment prime did not enhance within-session extinction learning or between session extinction. Furthermore, this pattern of results was not moderated by individual differences in attachment style, suggesting that the attachment priming effects held for participants with varying levels of attachment security.

These results extend on a previous report of attachment priming enhancing the extinction and preventing relapse of fear [12]. The current study adds to this earlier finding because it demonstrates that the beneficial impact of attachment priming extends beyond the influence of positive mood; that is, it appears that it is the sense of ‘felt security’ and ‘safety’ provided by heightening awareness of attachments that augments extinction [28]. Historically, safety signals present during extinction learning have been found to protect rather than enhance extinction [29]. This means that while safety signals during extinction lead to reductions in fear responding, once removed in a long-term test, they generally lead to more relapse of fear. Perhaps the positive control prime could be considered to have demonstrated this ‘protection from extinction’ effect, as it led to relapse of fear during reinstatement. To truly conclude this, a no-prime group would be needed in future studies. Nonetheless, the attachment prime seemed to reverse any deficit here, because it demonstrated less relapse of fear. As such, the current finding provides strong support for the conclusion that attachment primes serve as “unique safety signals” that enhance the long-term inhibitory learning acquired through extinction [30].

Our findings diverged from the previous study on attachment and extinction learning in two ways. First, unlike the Hornstein et al. (2017) [12] study, we found that the attachment prime did not inhibit fear during extinction. The current results indicated that the startle response did not extinguish to the CS+ relative to the CS− during the first or second session of extinction. This difference could be attributed to several factors. First, the two studies differed on the protocol used for fear acquisition and fear extinction. The former used only four paired presentations of the CS+ with shock during acquisition and extinction and utilized a 100% reinforcement protocol, which purportedly leads to very rapid extinction learning [6]. Our study used 16 trials of the CS+ in acquisition and extinction with a partial reinforcement schedule, which is shown to slow extinction learning [6]. It is possible that we needed to increase the number of trials during the extinction blocks to observe more pervasive extinction learning [31].

Second, the absence of priming effects within extinction in our study could have been driven by the type of control group used. Our control group was designed to match the priming conditions on positive mood, as this is a known variable that affects extinction learning [15]. In fact, our control was rated by participants as more “exciting” and as equally “positive” relative to the attachment group, indicating that any effects we report here are held above and beyond the role of positive mood and arousal. The Hornstein et al. (2017) [12] study used images of strangers as their control. Previous studies have shown that these are generally rated as less positive than images of attachment figures [17]. It is possible that the previous reports of attachment images inhibiting fear during extinction could be explained by greater activation of positive mood and/or arousal within the learning phase. This interpretation is supported by evidence that positive mood inducement via imagery [15] or counterconditioning with auditory cues [14] enhances within-session extinction learning.

Historically, safety signals (or positive mood inducement) present during extinction learning have been found to protect rather than enhance long-term extinction retention [29]. Perhaps the positive control prime could be considered to have demonstrated this ‘protection from extinction’ effect, as it led to relapse of fear during reinstatement. To truly conclude this, a no-prime group would be needed in future studies. Nonetheless, the attachment prime seemed to reverse any deficit here, showing less relapse of fear. As such, we can conclude consistently with Hornstein and Eisenberger [30] that these attachment primes (whether visual or imaginative) serve as “unique safety signals” that enhance the long-term inhibitory learning acquired through extinction. In fact, our data here would be consistent with the conclusion that the attachment prime enhanced ‘safety learning’, in that the effects were held equally for both the fear (CS+) and safety (CS−) stimuli.

There are methodological issues in the current study that need to be acknowledged. First, to limit disruption of the prime activation, we did not re-administer any post-manipulation check on levels of positive mood. Future studies could use such post-manipulation checks to index whether the groups were truly equated on positive mood after the manipulation [15]. Second, the primes were rated differently in terms of vividness. It is understandable that imagining a hypothetical situation in the positive prime condition was less vividly imagined than the attachment group’s imagery of their familiar attachment figure. It should be noted that these differences did not explain the effects of the prime on fear learning. Nonetheless, future studies should attempt to control for these factors in ways that primes differ only on the extent to which they direct participants to be aware of attachment figures. Third, it is possible that the study was not adequately powered to detect moderation effects involving individual differences in attachment style; at this stage it is unknown the potential strength of any effect of attachment style differences, and so estimating the required sample size is difficult. Fourth, the sample consisted of a non-clinical population, and a convenience sample that tended to be primarily a younger female demographic. This has obvious repercussions for the generalizability of our results, and in particular may result in an underrepresentation of individuals high on attachment anxiety or avoidance. Research has found that clinical samples tend to have higher rates of insecure attachment styles (73%) than non-clinical samples (42%) [32].

These limitations notwithstanding, the current study has significant translational potential. Here we report that brief activation of a secure attachment figure prior to undergoing extinction learning can reduce susceptibility to showing a return of fear at a long-term test. If these results can be translated to a clinical sample, they have the potential to find that a brief attachment intervention prior to undergoing exposure therapy can enhance long-term effectiveness of the most common strategy used to manage anxiety conditions.

## Supplementary information

Supplementary Material

Supplementary Figure

## References

[1] Bandelow B, Michaelis S (2015). Epidemiology of anxiety disorders in the 21st century. Dialogues Clin Neurosci.

[2] Carpenter JK, Andrews LA, Witcraft SM, Powers MB, Smits JAJ, Hofmann SG (2018). Cognitive behavioral therapy for anxiety and related disorders: a meta-analysis of randomized placebo-controlled trials. Depress Anxiety.

[3] Springer KS, Levy HC, Tolin DF (2018). Remission in CBT for adult anxiety disorders: a meta-analysis. Clin Psychol Rev..

[4] Butler AC, Chapman JE, Forman EM, Beck AT (2006). The empirical status of cognitive-behavioral therapy: a review of meta-analyses. Clin Psychol Rev..

[5] Johnson LR, McGuire J, Lazarus R, Palmer AA (2012). Pavlovian fear memory circuits and phenotype models of PTSD. Neuropharmacology.

[6] Bouton ME (2004). Context and behavioral processes in extinction. Learn Mem.

[7] Lebois LAM, Seligowski AV, Wolff JD, Hill SB, Ressler KJ (2019). Augmentation of extinction and inhibitory learning in anxiety and trauma-related disorders. Annu Rev Clin Psychol..

[8] Mikulincer M & Shaver PR. Attachment in adulthood: structure, dynamics, and change. 2nd ed. Guildford; 2016.

[9] Hornstein EA, Eisenberger NI (2017). Unpacking the buffering effect of social support figures: social support attenuates fear acquisition. PLoS ONE.

[10] Toumbelekis M, Liddell BJ, Bryant RA (2018). Thinking of attachment figures blocks differential fear conditioning. Soc Cogn Affect Neurosci.

[11] Toumbelekis M, Liddell BJ, Bryant RA (2021). Secure attachment primes reduce fear consolidation. Depress Anxiety.

[12] Hornstein EA, Haltom KEB, Shirole K, Eisenberger NI (2017). A unique safety signal: social-support figures enhance rather than protect from fear extinction. Clin Psychol Sci..

[13] Kang S, Vervliet B, Engelhard IM, van Dis EA, Hagenaars MA (2018). Reduced return of threat expectancy after counterconditioning verus extinction. Behav Res Ther..

[14] Raes AK, De Raedt R (2012). The effect of counterconditioning on evaluative responses and harm expectancy in a fear conditioning paradigm. Behav Ther..

[15] Zbozinek TD, Holmes EA, Craske MG (2015). The effect of positive mood induction on reducing reinstatement of fear: Relevance for long term outcomes of exposure therapy. Behav Res Ther..

[16] Bryant RA, Datta S (2019). Reconsolidating intrusive distressing memories by thinking of attachment figures. Clin Psychol Sci..

[17] Mikulincer M, Hirschberger G, Gillath O (2001). The affective component of the secure base schema: Affective priming with representations of proximity maintenance. J Pers Soc Psychol.

[18] Lovibond PF, Lovibond SH (1995). The structure of negative emotional states: comparison of the Depression Anxiety Stress Scales (DASS) with the Beck Depression and Anxiety Inventories. Behav Res Ther..

[19] Antony MM, Bieling PJ, Cox BJ, W. EM, Swinson RP (1998). Psychometric properties of the 42-item and 21-item versions of the Depression Anxiety Stress Scales in clinical groups and a community sample. Psychol Assess..

[20] Brennan KA, Clark CL, Shaver PR (1998). Attachment theory and close relationships.

[21] Sibley CG, Fischer R, Liu JH (2005). Reliability and validity of the revised experiences in close relationships (ECR-R) self-report measure of adult romantic attachment. Pers Soc Psychol Bull..

[22] Marks DF (1973). Visual imagery differences in the recall of pictures. Br J Psychol.

[23] Lonsdorf TB, Menz MM, Andreatta M, Fullana MA, Golkar A, Haaker J (2017). Don’t fear ‘fear conditioning’: Methodological considerations for the design and analysis of studies on human fear acquisition, extinction, and return of fear. Neurosci Biobehav Rev..

[24] Blumenthal TD, Cuthbert BN, Filion DL, Hackley S, Lipp OV, van Boxtel A (2005). Committee report: Guidelines for human startle eyeblink electromyographic studies. Psychophysiology.

[25] Grillon C, Ameli R (2001). Conditioned inhibition of fear-potentiated startle and skin conductance in humans. Psychophysiology.

[26] Hermans D, Dirikx T, Vansteenwegen D, Baeyens F, van den Bergh O, Eelen P (2005). Reinstatement of fear responses in human aversive conditioning. Behav Res Ther..

[27] Norrholm SD, Jovanovic T, Vervliet B, Myers KM, Davis M, Rothbaum BO (2006). Conditioned fear extinction and reinstatement in a human fear-potentiated startle paradigm. Learn Mem..

[28] Carnelley KB, Rowe AC (2010). Priming a sense of security: What goes through people’s minds?. J Soc Pers Relatsh.

[29] Lovibond PF, Davis NR, O’Flaherty AS (2000). Protection from extinction in human fear conditioning. Behav Res Ther..

[30] Hornstein EA, Eisenberger NI (2018). A social safety net: developing a model of social-support figures as prepared safety stimuli. Curr Direct Psychol Sci..

[31] Phelps EA, Delgado MR, Nearing KI, LeDoux JE (2004). Extinction learning in humans: Role of the amygdala and vmPFC. Neuron.

[32] Bakermans-Kranenburg MJ, van Ijzendoorn MH (2009). The first 10,000 Adult Attachment Interviews: distributions of adult attachment representations in clinical and non-clinical groups. Attach Hum Dev.

