# Supplementary material for: Secure attachment priming protects against relapse of fear in Young adults
Source: Transl Psychiatry. 2021 Nov 13;11:584. doi: 10.1038/s41398-021-01715-x (PMC8590684; doi:10.1038/s41398-021-01715-x)
Supplement: Supplementary file 1 — Supplementary Material [file 41398_2021_1715_MOESM1_ESM.docx]

**Supplementary Methods**

***Startle Measurement***

Two 4mm Ag-AgCl electrodes were filled with electrolyte gel and the electrodes were placed approximately 1 cm below the left pupil and below the external canthus. The ground electrode was placed behind the neck. Impendance level was kept below 5 KΩ. The raw EMG was amplified and digitized at 1000 Hz using the AD Instruments Dual Bio Amp (FE135, AD Instruments, Sydney). The signal was then filtered (band-pass = 28-500 Hz), rectified and smoothed with a running average window of 25ms (LabChart, AD Instruments).

***Procedure***

Participants were verbally instructed as follows prior to the pre-conditioning phase.  They were told that they should “indicate on the response meter how likely they expect to receive a shock at the end of each colored square.  On every trial you may or may not be shocked, but if you pay attention to the different colored squares, you may be able to predict when the shocks will be delivered”.  Prior to extinction, instructions mentioned that participants would “continue the same task as before the break” and they were instructed to “continue giving your responses on the meter as to how likely they expect to receive a shock on each trial, remembering that on each trial you may or may not be shocked”.

***Data Reduction***

The magnitude of the blink reflex was calculated as the peak value within 21-120ms following the startle stimulus relative to a baseline, taken as the average EMG response during the 20ms preceding the onset of the startle probe. Trials on which the baseline was too noisy, with excessive activity during the 20ms following the probe relative to baseline, were removed (consistent with Grillon & Ameli, 2001). Trials which gave negative startle scores were replaced with a 0 response (consistent with Lonsdorf, et. al., 2017). Differential scores were calculated as the difference between blinks occurring during CS trials compared to blinks during the startle alone trials. This was to remove excessive variance arising from individual differences in startle levels as well as to remove some of the effect of habituation across time (Lonsdorf, et. al., 2017).

***Post-hoc Discrimination Analyses***

The exclusion of non-learners from the main analyses in this experiment raised the question of the mechanisms underlying the learning process in this subset of participants. In particular, if this subgroup happened to display the documented phenomenon of fear generalization, where they might have been showing fear to both the CS+ and CS-, rather than fear discrimination, i.e. greater fear to the CS+ than the CS-. In order to assess these questions, participants startle scores were calculated into ‘fear discrimination’ ratio scores according to the formula: (CS+ minus CS-)/CS-; and ‘fear generalization’ scores according to the formula: (CS- minus startle alone baseline)/startle alone baseline. The former assessed the proportion of fear that was uniquely discriminating to the CS+ cue that predicted shock, and the latter was to quantify how much fear was ‘generalized’ to the CS- (not predictive of shock) over and above baseline startle levels.

Analyses conducted on these scores looked to compare between the groups classified as ‘learners’ and ‘non-learners’ (as explained in the main manuscript) in their levels of fear discrimination and fear generalization. Also, these ratio scores were examined against the threshold scores of 0 for ‘fear discrimination’ – indicating successful CS+ fear discrimination.

An overall, repeated-measures ANOVA was conducted on the repeated-measures variable of phase (including conditioning, extinction, recall, and reinstatement), with the between-group variables of learners (learners vs. non-learners) and group (attachment vs. positive control group) for the ‘fear discrimination’ and ‘fear generalization’ ratio scores respectively.

**Results**

Analysis of the sample of participants including those who met exclusion criteria as startle non-learners (n=14) is included here. This sample included a total of 95 participants (65 females, mean age = 19.9 years, *SD*=2.33) with 49 participants in the attachment group and 46 in the control group. Demographic characteristics and questionnaire scores did not significantly differ between groups (all *p*’s>0.05). Baseline phases, including habituation and pre-conditioning reported no differences between groups [Habituation: *F*(1,93)=0.56,*p*=.458*, η^2^_p_*=0.006; Pre-conditioning: *F*(1,93)=0.05,*p*=.831*, η^2^_p_*<0.001]. During the conditioning phase, there remained a significant main effect of CS (*F*(1,93)=45.87,*p*<.001*, η^2^_p_*=0.330) indicating successful acquisition of fear learning. Furthermore, as reported in the manuscript, startle scores extinguished to both the CS+ and CS- in the extinction (*F*(1,93)=31.07,*p*<.001*, η^2^_p_*=0.250) and extinction recall phases (*F*(1,93)=25.68,*p*<.001*, η^2^_p_*=0.216). From the recall to reinstatement phase, the 3-way interaction between phase, cs and group remained significant (*F*(1,93)=5.32,*p*=.023*, η^2^_p_*=0.054), and simple effects showed that the reinstatement effect (phase by cs interaction) was larger for the control group (*F*(1,45)=1.51,*p*=.225*, η^2^_p_*=0.033) than the attachment group (*F*(1,48)=0.43,*p*=.517*, η^2^_p_*=0.009). Furthermore, there was lower overall levels of startle to both the CS+ and CS- during the reinstatement phase for the attachment group (*F*(1,93)=5.26,*p*=.024*, η^2^_p_*=0.054).

Due to the inclusion of participants with negative scores during fear learning, percent recall ratio scores became uninterpretable, therefore all reported regression and moderation effect analyses reported in the manuscript could not be carried out on this sample of participants.

***Post-hoc Discrimination Analyses***

For ‘fear discrimination’ scores, there was a significant overall main effect of learners [*F*(1,91)=12.38, *p*=.001, *η^2^_p_*=0.116], where averaged across the phases, participants classified as ‘learners’ showed greater fear discrimination to the CS+ relative to the CS- cue than ‘non-learners’. This effect was expected in the conditioning phase, however, examining simple effects illustrated that those classified as non-learners failed to demonstrate a reliable fear discrimination effect during any of the other phases during the experiment (i.e. not significantly greater than the threshold of 0). See Table S3 for a summary of the statistics.

There were no group effects, whereby the attachment and positive prime group did not demonstrate on average different levels of fear-discrimination scores, nor was there an interaction between group and their ‘learner’ classification on these scores (main effects *p’s*>0.63).

For ‘fear generalization’ scores, there were no main effects of phase, ‘learner’ classification or group status (all *p’s*>0.22). See Table S4 for a summary of the statistics. However, all scores were significantly greater than the threshold of 0, indicating, as expected, that all participants had greater startle to the CS- cue compared to startle-alone baseline trials [*F*(1,91)=12.2, *p*=.001, *η^2^_p_*=0.118].

In summary, these results indicate that that the participants excluded as ‘non-learners’ were not differentiating between CS+ and CS- in their startle scores across any of the phases in the experiment, nor where they responding proportionally different to the CS- than the CS+ or startle-alone baseline trials as might be expected if they were showing generalized fear.

**Supplementary Data**

*Table S1.* Post-hoc fear-discrimination analyses. Fear-discrimination scores were calculated according to the formula (CS+ minus CS-)/CS-. Simple effects analyses were conducted for participants classified as ‘learners’ (n=) and ‘non-learners’ (n=12) in separate MANOVAs including the different phases of the experiment (conditioning, extinction, recall, reinstatement) as within-group variables and the prime groups as a between-group variable. Note that the intercept variable examines differences from the threshold of 0 value, indicating a significant discrimination between CS+ and CS-.

| Source | Measure | *M* | *SD* | *F*(1,79) | *p* | *η^2^_p_* |
| --- | --- | --- | --- | --- | --- | --- |
| ‘Learners’ | Conditioning | 0.50 | .551 | 1.22 | .273 | 0.015 |
| (Corrected for | Extinction | 0.65 | .957 | 0.05 | .825 | 0.001 |
| Group effect) | Recall | 0.47 | .696 | 0.05 | .833 | 0.001 |
|  | Reinstatement | 0.66 | .956 | 0.002 | .961 | <.001 |
| ‘Learners’ | Conditioning |  |  | 65.30 | <.001 | 0.453 |
| Intercept | Extinction |  |  | 36.45 | <.001 | 0.316 |
|  | Recall |  |  | 36.58 | <.001 | 0.317 |
|  | Reinstatement |  |  | 37.64 | <.001 | 0.323 |
| Subgroup | Measure | *M* | *SD* | *F*(1,12) | *p* | *η^2^_p_* |
| ‘Non-Learners’ | Conditioning | -0.12 | .107 | 0.13 | .724 | 0.011 |
| (Corrected for | Extinction | 0.20 | .358 | 0.25 | .625 | 0.021 |
| Group effect) | Recall | 0.01 | .288 | 2.49 | .140 | 0.172 |
|  | Reinstatement | -0.09 | .288 | 0.08 | .782 | 0.007 |
| ‘Non-Learners’ | Conditioning |  |  | 15.19 | .002 | 0.559 |
| Intercept | Extinction |  |  | 3.58 | .083 | 0.230 |
|  | Recall |  |  | 0.003 | .958 | <.001 |
|  | Reinstatement |  |  | 1.23 | .288 | 0.093 |

*Table S2.* Post-hoc fear-generalization analyses. Fear-generalization scores were calculated according to the formula (CS- minus startle-alone)/startle-alone trials. Simple Effect analyses were conducted for participants classified as ‘learners’ (n=) and ‘non-learners’ (n=12) in separate MANOVAs including the different phases of the experiment (conditioning, extinction, recall, reinstatement) as within-group variables and the between-group variable of the prime groups. N.B. the intercept variable looks at differences from the threshold of 0 value, indicating a significant discrimination between CS- and startle-alone trials.

| Source | Measure | *M* | *SD* | *F*(1,79) | *p* | *η^2^_p_* |
| --- | --- | --- | --- | --- | --- | --- |
| ‘Learners’ | Conditioning | 0.81 | 1.51 | 0.07 | .794 | 0.001 |
| By Group effect | Extinction | 1.75 | 6.34 | 1.17 | .284 | 0.015 |
|  | Recall | 0.88 | 1.57 | 0.87 | .353 | 0.011 |
|  | Reinstatement | 0.75 | 1.21 | 0.23 | .633 | 0.003 |
| ‘Learners’ | Conditioning |  |  | 22.96 | <.001 | 0.225 |
| Intercept | Extinction |  |  | 6.11 | .016 | 0.072 |
|  | Recall |  |  | 25.11 | <.001 | 0.241 |
|  | Reinstatement |  |  | 30.98 | <.001 | 0.282 |
| Subgroup | Measure | *M* | *SD* | *F*(1,12) | *p* | *η^2^_p_* |
| ‘Non-Learners’ | Conditioning | 1.34 | 1.30 | 0.11 | .745 | 0.009 |
| By Group effect | Extinction | 0.66 | 0.74 | 0.13 | .729 | 0.010 |
|  | Recall | 0.81 | 0.59 | 0.36 | .562 | 0.029 |
|  | Reinstatement | 0.81 | 0.65 | 1.28 | .280 | 0.096 |
| ‘Non-Learners’ | Conditioning |  |  | 22.96 | <.001 | 0.225 |
| Intercept | Extinction |  |  | 6.11 | .016 | 0.072 |
|  | Recall |  |  | 25.11 | <.001 | 0.241 |
|  | Reinstatement |  |  | 30.98 | <.001 | 0.282 |

*Table S3.* Range and distribution of self-report scales.

| Measure | *Range* | *Minimum* | *Maximum* | *Skewness* | *Kurtosis* |
| --- | --- | --- | --- | --- | --- |
| DASS (Depression) | 13 | 0 | 13 | 1.37 | 1.06 |
| DASS (Anxiety) | 14 | 0 | 14 | 1.72 | 3.97 |
| DASS (Stress) | 15 | 0 | 15 | 0.94 | 1.16 |
| VVIQ | 4 | 1 | 5 | -0.83 | 2.99 |
| ECR (Anxiety) | 4.28 | 1.28 | 5.56 | 0.14 | 0.03 |
| ECR (Avoidance) | 4.72 | 1 | 5.72 | 0.37 | -0.41 |

*Table S4.* Moderation analyses with ratings of prime on the dependent variables of percent recall scores at the reinstatement test.

| Source | *B* | *SE B* | *b* | *t* | *p* |
| --- | --- | --- | --- | --- | --- |
| (Constant) | -50.62 | 46.24 |  | -1.09 | .277 |
| Prime | 85.54 | 29.20 | 0.32 | 2.93 | .004 |
| Positive ratings | -37.97 | 45.06 | -0.33 | -0.84 | .402 |
| Positive x Prime | 24.74 | 26.34 | 0.36 | 0.94 | .351 |
| (Constant) | -52.33 | 47.68 |  | -1.10 | .276 |
| Prime | 85.78 | 30.19 | 0.32 | 2.84 | .006 |
| Excited ratings | 2.12 | 34.31 | 0.02 | 0.06 | .951 |
| Excited x Prime | -1.23 | 21.38 | -0.02 | -0.06 | .954 |
| (Constant) | -62.46 | 72.37 |  | -0.86 | .391 |
| Prime | 100.01 | 42.57 | 0.37 | 2.35 | .021 |
| Closeness ratings | -18.58 | 43.64 | -0.30 | -0.43 | .672 |
| Closeness x Prime | 15.11 | 23.48 | 0.42 | 0.64 | .522 |
| (Constant) | -40.08 | 48.78 |  | -0.82 | .414 |
| Prime | 78.15 | 30.47 | 0.29 | 2.57 | .012 |
| Vividness ratings | -29.30 | 74.01 | -0.17 | -0.40 | .693 |
| Vividness x Prime | 7.44 | 42.47 | 0.07 | 0.18 | .861 |

*Note.* Positive, *R*^2^ = .128; Excited, *R*^2^ = .117; Closeness, *R*^2^ = .136; Vividness, *R*^2^ = .125

*Figure S1.* Subjective ratings of the prime (group means +SEMs). Participants were asked to rate on a 1-10 Likert scale how “positive”, “excited”, “close to others” their chosen person/hypothetical situation made them feel prior to engaging in the mental imagery. Then they were asked to rate on a 1-5 Likert scale how “vivid” the mental imagery was following the imagination exercise.
