# Supplementary figures and images for: Secure attachment priming protects against relapse of fear in Young adults
Source: Transl Psychiatry. 2021 Nov 13;11:584. doi: 10.1038/s41398-021-01715-x (PMC8590684; doi:10.1038/s41398-021-01715-x)

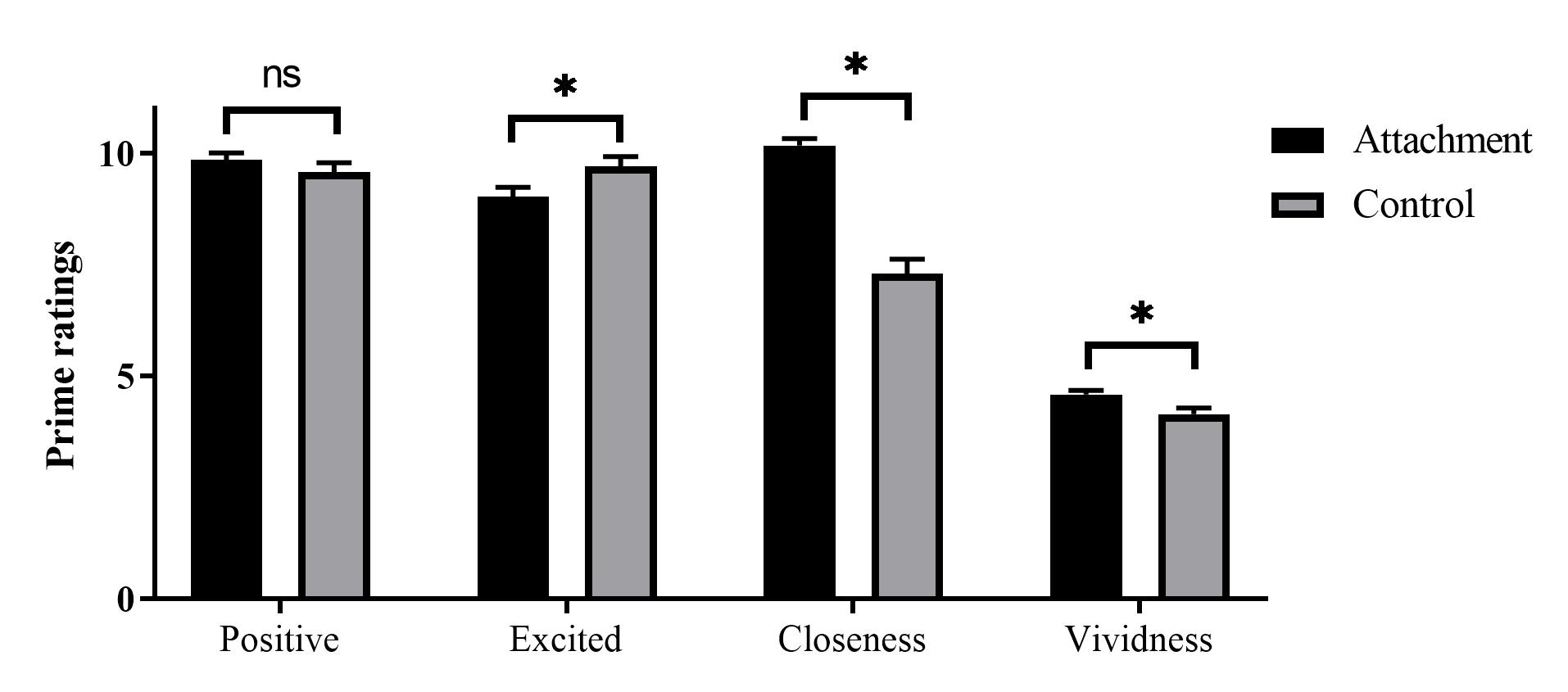

Supplement: Supplementary file 2 — Supplementary Figure [file 41398_2021_1715_MOESM2_ESM.jpg]
